# Supplementary material for: Bioconversion of agro-industrial residues as a protein source supplementation for multiparous Holstein Thai crossbreed cows
Source: PLoS One. 2022 Sep 1;17(9):e0273916. doi: 10.1371/journal.pone.0273916 (PMC9436144; doi:10.1371/journal.pone.0273916)
Supplement: Attachment — Submitted filename: Response-to-Reviwer_CS-e.docx [file pone.0273916.s001.docx]

Tropical Feed Resources Research and Development Center (TROFREC)

Department of Animal Science

Faculty of Agriculture

Khon Kaen University

Khon Kaen 40002

Thailand

Tel./Fax 6643202368

Dear Prof. Dr. Mahmoud A.O. Dawood, Academic Editor of PLOS ONE,

Submission ID: PONE-D-22-12915R1

Type of manuscript: Article

Title: Bioconversion of agro-industrial residues as a protein source supplementation for multiparous Holstein Thai crossbreed cows

Authors: Suriyapha et al.

We appreciate all of the guidance and suggestions from the one reviewers and Journal office, as you gave us a great chance to revise the manuscript and keep improving on our work. Above all, the authors thought that all of the issues raised were really helpful, and they have taken into account the majority of the revisions where they were recommended in order to prepare the text for potential publication. As a result, we hope that the current work is ready for publication.

Using the "Track Changes" feature in Microsoft Word, all of those that were corrected and amended are now visible in the manuscript.

With the aforementioned details, we would like to kindly request your attention as we resubmit our manuscript for potential publication in PLOS ONE.

Once more, we appreciate your kind consideration and assistance.

Sincerely yours,

Assoc. Prof. Dr. Anusorn Cherdthong

Corresponding author

Contact: [Anusornc@kku.ac.th](mailto:Anusornc@kku.ac.th)

**Response to Journal Requirements:**

When submitting your revision, we need you to address these additional requirements.

**Response: Thank you, we have done it. Please see below.**

1. Please ensure that your manuscript meets PLOS ONE's style requirements, including those for file naming. The PLOS ONE style templates can be found at

https://journals.plos.org/plosone/s/file?id=wjVg/PLOSOne_formatting_sample_main_body.pdf and

https://journals.plos.org/plosone/s/file?id=ba62/PLOSOne_formatting_sample_title_authors_affiliations.pdf

**Response: Thank you. We have modified according to the style requirement. Please see in manuscript.**

2. Thank you for stating the following in the Acknowledgments Section of your manuscript:

“-CS, AC

-RRI-PHD62I0021

-Research and Researchers for Industries

-http://rri.trf.or.th/bs_detail.asp

-Funders did not play any role in the study design, data collection and analysis, decision to publish, or preparation of the manuscript.”

We note that you have provided additional information within the Acknowledgements Section that is not currently declared in your Funding Statement. Please note that funding information should not appear in the Acknowledgments section or other areas of your manuscript. We will only publish funding information present in the Funding Statement section of the online submission form.

Please remove any funding-related text from the manuscript and let us know how you would like to update your Funding Statement. Currently, your Funding Statement reads as follows:

“The authors would like to express our sincere thanks to the Animal Feed Inter Trade 398 Co., Ltd and Thailand Science Research and Innovation (TSRI) through the Research and 399 Researcher for Industry (RRi) program (contract grant PHD62I0021) for the scholar funding 400 and research grant”

Please include your amended statements within your cover letter; we will change the online submission form on your behalf.

**Response: Thank you. The acknowledgments have been modified as:**

**The authors would like to express our sincere thanks to the Thailand and Tropical Feed Resources Research and Development Center (TROFREC), Department of Animal Science, Faculty of Agriculture, Khon Kaen University (KKU) for the use of the research facilities and making this work possible. The authors acknowledge the KSL Green Innovation Public Co., Ltd, Sukkhee Farm, Thailand. Moreover, the authors are sincerely grateful to acknowledge J. P. Schoonmaker (Associate Professor in the Department of Animal Sciences, Purdue University, USA) for his help in recommending, suggesting, and proofreading our manuscript.**

**In addition, the funding has been removed from the manuscript and please publish funding information as**

**Funding**

**This work was supported by Animal Feed Inter Trade Co., Ltd, Thailand Science Research and Innovation (https://www.tsri.or.th) through the Research and Researcher for Industry (RRi) (http://rri.trf.or.th/bs_detail.asp) program (contract grant PHD62I0021), Research Program on the Research and Development of Winged Bean Root Utilization as Ruminant Feed, Increase Production Efficiency and Meat Quality of Native Beef and Buffalo Research Group and Research and Graduate Studies, Khon Kaen University (KKU), and Office of National Higher Education Science Research and Innovation Policy Council (https://www.nxpo.or.th/) through the Program Management Unit for Competitiveness (PMUC) (https://www.nxpo.or.th/) (contract grant C10F640078). Funders did not play any role in the study design, data collection and analysis, decision to publish, or preparation of the manuscript.**

3. Please review your reference list to ensure that it is complete and correct. If you have cited papers that have been retracted, please include the rationale for doing so in the manuscript text, or remove these references and replace them with relevant current references. Any changes to the reference list should be mentioned in the rebuttal letter that accompanies your revised manuscript. If you need to cite a retracted article, indicate the article’s retracted status in the References list and also include a citation and full reference for the retraction notice.

**Response: We have checked and modified.**

**Response to Reviewer 1**

Introduction

1. Need to provide more information to justify why using CWYW to alternate SBM - any economical value?

**Response: Thank you for your suggestion. We have provided as “When comparing economic values, CWYW was cheaper than SBM by 50% per kilogram (0.31 USD vs. 0.61 USD).” Please see in line 66-67. In addition, we have provided detail of feed cost and CWYW cost in the results section and Table 1. Please see in manuscript.**

Materials and Methods

1. Please mention yeast species

**Response: Thanks for your suggestion.** **Present study we used *Saccharomyces cerevisiae*. We have added as “A by-product of ethanol manufacturing called yeast waste, which includes inoculants of the yeast (*Saccharomyces cerevisiae*), was obtained from Green Innovation Public Company Limited (KGI) a subsidiary of Khon Kaen Sugarcane Industry Company Limited in Nam Phong district, Khon Kaen province, Thailand.”** **Please see in line** **82-85.**

**Thanks so much for the valuable comments!**
